# The effect of sukha pranayama on anxiety in patients undergoing coronary angiography: a single -blind randomized controlled trial

**DOI:** 10.15171/jcvtr.2016.34

**Published:** 2016-12-30

**Authors:** Maryam Mobini Bidgoli, Mohsen Taghadosi, Hamidreza Gilasi, Alireza Farokhian

**Affiliations:** ^1^Faculty of Nursing and Midwifery, Kashan University of Medical Sciences, Kashan, Iran; ^2^Department of Medical Surgical, Fculty of Nursing and Midwifery, Kashan University of Medical Sciences, Kashan, Iran; ^3^Department of Biostatistics and Epidemiology, Faculty of Health, Kashan University of Medical Sciences, Kashan, Iran; ^4^Department of Cardiology, Faculty of Medicine, Kashan University of Medical Sciences, Kashan, Iran

**Keywords:** Coronary Angiography, Sukha Pranayama, Anxiety

## Abstract

***Introduction:*** Anxiety is among the most common problems experienced by coronary angiography (CA) candidates. Different modalities are used to manage anxiety. This study sought to examine the effects of a pranayama exercise on CA candidates’ anxiety.

***Methods:*** This double-blind randomized controlled trial was undertaken in 2015 on 80 eligible patients. The patients were randomly allocated to a control and an experimental group. Before undergoing angiography, patients in the experimental group performed sukha pranayama exercises. They were trained to breathe slowly and rhythmically at a rate of ten breathing per minute for five consecutive minutes. Patients in the control group only received routine preangiography care. Data collection tools were a demographic questionnaire and the Spielberger State Anxiety Inventory. The level of patients’ anxiety in both groups was measured before, half an hour after, and one hour after the intervention. The data were analyzed through doing the independent-sample t and the chi-square tests.

***Results:*** Before the intervention, the mean of anxiety score in the experimental group was 53.37, which significantly decreased to 40.75 after the intervention (*P* = 0.0001). In the control group, the mean of anxiety score decreased from 54.27 to 51.4. This decrease was not statistically significant. Moreover, between-group comparisons revealed significant differences between the groups regarding between-measurement mean differences of anxiety score (*P* < 0.01).

***Conclusion:*** Sukha pranayama is effective in alleviating CA candidates’ anxiety.

## Introduction

In recent years, the use of diagnostic invasive procedures such as coronary angiography (CA) has increased due to an increase in the prevalence of cardiovascular disease and advances in medical technology. Currently, more than one million cardiac catheterizations CAs are performed each year in the United States.^1^ Patients who are candidate for undergoing CA usually experience unfamiliar conditions and environments. Besides, they are physically separated from their families and may have not adequate knowledge about medical interventions, hospitalization-related costs, and the likelihood of experiencing pain and anesthesia. Consequently, they may suffer from stress, anxiety, and even emotional shock.^2,3^ Different studies have shown that more than 80% of CA candidates are anxious and experience considerable stress before undergoing CA.^1^

Pre-CA anxiety is inevitable. Severe anxiety can considerably affect different bodily organs, particularly the heart.^4^ It triggers a set of physiologic and biochemical responses, activates the sympathetic nervous system, and precipitates the release of epinephrine and norepinephrine. Thereby, it increases blood pressure, heart rate, respiratory rate, cardiac workload, myocardial oxygen demand, and the risk for developing myocardial ischemia and cardiac dysrhythmia during CA.^1^ Therefore, effective anxiety reduction is of paramount importance to CA candidates.

CA-related anxiety can be reduced both pharmacologically and non-pharmacologically. Pharmacological anxiety reduction mainly includes the use of benzodiazepines. However, pharmacological anxiolytic agents are usually short-acting and produce different side effects. Consequently, non-pharmacological anxiety reduction modalities such as complementary therapies have received special attention during recent years.^5^ The use of complementary therapies is progressively increasing in both developed and developing countries. The results of a local study in Iran showed that 80% of patients were interested in receiving complementary therapies from their physicians.^6^ Complementary therapies include a wide range of modalities such as music therapy, herbal medicines, reflexology, and yoga.^7^

Different studies have investigated the anxiolytic effects of complementary therapies among CA candidates. For instance, Vardanjani et al found foot reflexology effective in reducing CA candidates’ anxiety.^8^ However, Astley et al found that audiovisual informational aids had no significant effects on anxiety among patients undergoing CA.^9^ Besides, Taylor-Piliae reported that music therapy and sensory interventions did not reduce Chinese patients’ anxiety before undergoing CA.^10^

One of the complementary therapies which is useful to improve individuals’ physical, mental, and psychological health status is yoga exercises.^11^ The root of yoga is planted in India. Yoga exercises fall into three main categories including physical exercises (asana), breath control (pranayama), and thinking (dhyana).^12^ Pranayama (or the control of breathing) is the traditional technique of slow and rhythmical breathing. It can increase parasympathetic tone, decrease sympathetic activity, and improve cardiovascular and respiratory functions. In addition, it helps improve physical and psychological health status through alleviating stress.^13^

Chiang et al implemented a relaxation-breathing intervention on 48 children with moderate-to-severe asthma. The children aged 6–14 and performed relaxation-breathing exercises 30 minutes a day for twelve weeks. This intervention was effective in alleviating the children’s anxiety.^14^ Hayama and Inoue also reported the positive effects of deep breathing on anxiety and fatigue among patients undergoing chemotherapy.^15^ Moreover, Ebnezar et al found that their integrated yoga intervention (consisting of meditation, pranayama, relaxation, and asana) had significant effects on anxiety among patients with knee osteoarthritis.^16^ Gupta et al also implemented a 3-month anuloma-viloma pranayama intervention for elderly people who suffered from anxiety. They measured their participants’ anxiety by using the Sinha’s anxiety test and reported that their intervention significantly alleviated elderly people’s anxiety.^17^ Nonetheless, Sendhilkumar et al implemented a 3-week pranayama-meditation intervention on a small sample of patients with Guillain-Barré syndrome and found it ineffective in alleviating anxiety.^18^

Previous studies have reported conflicting findings regarding the effectiveness of pranayama exercises. Besides, none of the previous studies have investigated the effectiveness of pranayama in alleviating CA-related anxiety. Therefore, this study was undertaken in order to examine the effects of a pranayama exercise on CA candidates’ anxiety.

## Materials and Methods

### Design

This double-blind randomized controlled trial was undertaken in 2015 under the permission of Kashan University of Medical Sciences, Kashan, Iran. The setting was the inpatient catheterization laboratory of Shahid Beheshti hospital, Kashan, Iran.

### Sample

**Figure 1:**
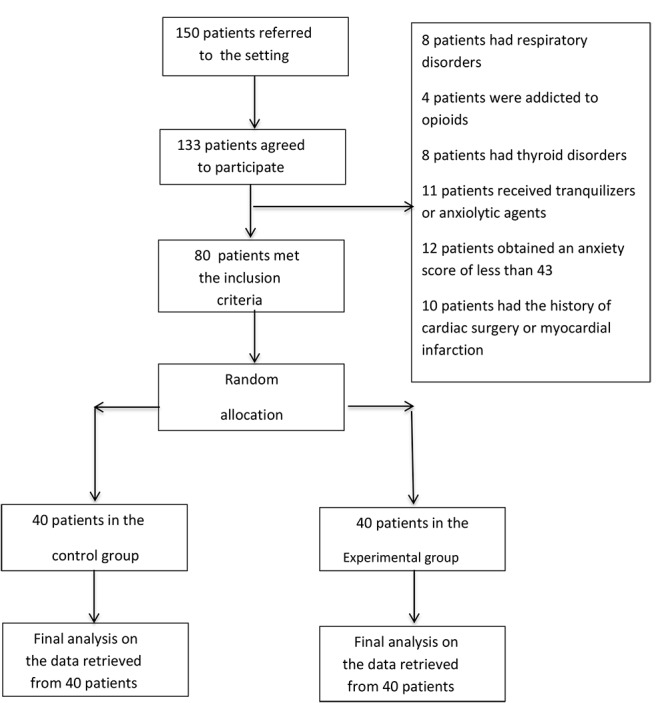
The process of sampling.

The sample size was calculated by using the formula for mean comparison in two or more independent samples. With a µ1 of 8, a µ2 of 5.9, a standard deviation of 3.5 (8), a confidence level of 0.95, and a power of 0.80, the sample size was determined to be 34. However, 40 patients were recruited to each study group in order to prevent probable attrition from affecting the power of the study. Consequently, all 150 eligible patients who referred to the study setting during the study were approached from which, 53 patients did not meet the inclusion criteria and seventeen cases refused to participate in the study. Therefore, 80 patients were consecutively recruited and randomly allocated to a control and an experimental group (Figure 1).

The inclusion criteria were undergoing CA for the first time, having a stable hemodynamic status before CA, having no dependence to addictive drugs, having received no tranquilizer or anxiolytic (such as propranolol) during the past 72 hours prior to the study, obtaining an anxiety score of 43 or more, and having no history of cardiac surgery, myocardial infarction, thyroid disorders, epilepsy, or respiratory conditions. Patients who were reluctant to stay in the study, were unable to endure the intervention, or developed vital signs instability were excluded.

### Instrument

Study instrument consisted of a demographic questionnaire and the Spielberger State Anxiety Inventory (SAI) which were completed on a self-reported basis. The items of the demographic questionnaire were age, gender, past medical history, and marital, educational, and employment status. The SAI is a well-known and standard anxiety assessment tool. It has 20 items which are scored from 1 (stand for ‘Not at all’) to 4 (‘Very much’), resulting in a total SAI score of 20 (lowest possible anxiety) to 80 (highest possible anxiety). The Persian version of the SAI is a valid and reliable instrument which has been used frequently for different populations in Iran. Dehghan-Nayeri and Bayani et al respectively reported a Cronbach alpha of 0.94 and 0.92 for the Persian SAI.^19,20^

### Intervention

Patients in the experimental group were trained to perform sukha pranayama breathing exercises as follows: “Close your eyes in order to be able to concentrate effectively. Breathe regularly and slowly through nostrils and by using inferior, medial, and superior parts of the lung in each inhalation and exhalation.” We attempted to control the participants’ breathing during the intervention through providing them with verbal instructions. This technique helped them to consciously use different parts of their lungs during sukha pranayama breathing exercises. Besides, they were asked to avoid thinking about any other thing than their breathing. We also instructed them to enter energy into their lungs during inhalation and expel tension, stress, and illness from their body at exhalation.^21^ Verbal instructions were provided in such a way that the rate of breathing was six respirations per minute. Moreover, the length of inhalation was deemed to be equal to the length of exhalation. Time measurements were made by using a chronometer. The intervention was implemented on each patient for five minutes and supervised by a cardiologist. On the other hand, patients in the control group received routine CA-related care services which included no breathing training. The level of patients’ anxiety in both groups was measured before (T1), half an hour after (T2), and one hour after the intervention (T3).

### Data analysis

The SPSS 16 was employed for data analysis. Between-group comparisons were made through running the independent-sample *t* and the chi-square tests. Moreover, the repeated measure analysis of variance (RM ANOVA) was used for making comparisons across the three measurement time points. The level of significance was set at less than 0.05.

## Results

The means of the patients’ age in the control and the sukha pranayama groups were 62.7 ± 6.28 and 55.5 ± 7.36, respectively. Despite random allocation of the patients to the treatments, the study groups differed significantly from each other regarding the patients’ age, educational status, and the history of hypertension. Therefore, these variables were entered into the analysis model as covariance. The groups did not differ significantly from each other in terms of gender, marital status, employment status, and the history of smoking, diabetes mellitus, and hyperlipidemia (Table 1).

**Table 1:** The frequency distribution of the participants’ demographic characteristics

** Characteristics**		** Group**		*** P***** value**
		** Sukha pranayama No. (%)**	** Control No. (%)**	
Gender	Male	18(45)	22(55)	0.37
	Female	22(55)	18(45)	
Age (y)	Mean ± SD	55.5±7.36	62.7±6.28	0.0001
Marital status	Single	3(7.5)	5(12.5)	0.46
	Married	37(92.5)	35(87.5)	
Educational status	Illiterate	11 (27.5)	14 (35)	0.04
	Primary	12 (30)	20 (50)	
	Guidance school	5 (12.5)	3 (7.5)	
	Diploma	10 (25)	1 (2.5)	
	Higher education	2 (0.5)	2 (0.5)	
Employment status	Employed	14 (35)	11 (27.5)	0.53
	Retired	7 (17.5)	11 (27.5)	
	Housewife	19 (47.5)	18 (45)	
Cigarette smoking	Yes	8(20)	10(25)	0.60
	No	32(80)	3(75)	
Reason behind CA	Resting chest pain	13 (32.5)	16 (40)	0.35
	A positive exercise test	1 (2.5)	2 (25)	
	Repetitive chest pain despite treatments	24 (24)	24 (24)	
Underlying conditions	Yes	27(67.5)	31(77.5)	0.32
	No	13(32.5)	9(22.5)	
Hypertension	Yes	16 (40)	25 (62.5)	0.04
Diabetes mellitus	Yes	16 (40)	21 (52.5)	0.26
Hyperlipidemia	Yes	21 (52.5)	20 (50)	0.82

**Figure 2:**
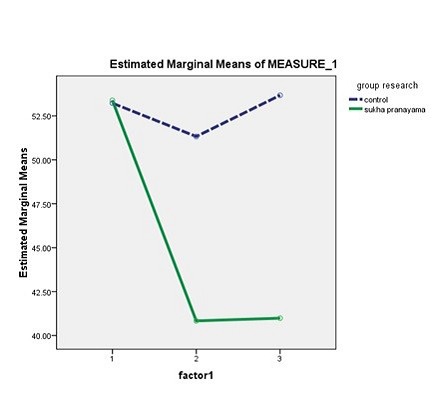
The mean anxiety score in both groups at T1, T2, and T3

Study findings showed that before the intervention, the difference between the groups concerning the mean score of anxiety was not statistically significant (*P* = 0.47; Table 2). In the experimental group, the mean score of anxiety at T2 and T3 was significantly lower than the corresponding pretest value by respectively 12.62 and 12.15 points (*P* = 0.0001). The amount of decrease in the anxiety mean score of the patients in the control group at T2 and T3 was 2.87 and 0.1, none of which was statistically significant (*P* = 0.87; Table 3). Moreover, between-group comparisons revealed that there were significant differences between the groups regarding T1-T2, T2-T3, and T1-T3 mean differences of the anxiety score (*P* < 0.05; Table 4)( Figure 2).

**Table 2:** Comparing the study groups regarding the mean score of anxiety

** Variable**		** Experimental**	** Control**	** 95% CI**	**P value (the independent-sample t test) **
		** Mean ± SD**	** Mean ± SD **		
Anxiety	Before (T1)	53.37 ± 6.11	5 ± 72.45	-1.58 , 3.38	0.47
	Half an hour after (T2)	80.7 ± 57.04	59.4 ± 4.15	7.92 , 13.37	0.0001
	One hour after (T3)	68.6 ± 22.14	26.5 ± 71.45	10.15 , 15.74	0.0001

**Table 3:** Mean scores anxiety of the study groups regarding T1-T2, T2-T3, and T1-T3

**Mean score of anxiety**	** Groups**					
	** Experimental**			** Control**		
	**T1-T2**	**T2-T3**	** T1-T3**	**T1-T2**	**T2-T3**	**T1-T3**
Mean ± SD	1.26±6.23	-0.47±4.15	1.21±6.83	2.86±3.63	-2.77±3.28	0.1±3.91
95% CI	10.36, 14.61	-1.80, 0.85	9.96, 14.33	1.71, 4	-3.82, -1.72	-1.15, 1.35
P value (paired-sample t test)	0.0001	0.47	0.0001	0.0001	0.0001	0.87

**Table 4:** Comparing the study groups regarding T1-T2, T2-T3, and T1-T3 mean differences of the anxiety score

	Groups	Mean±SD	P (t test)
T1-T2 mean difference	Control	-2.87 ± 3.63	0.0001
	Experimental	-12.62 ± 6.23	
T2-T3 mean difference	Control	2.77± 3.28	0.008
	Experimental	0.47 ± 4.15	
T1-T3 mean difference	Control	-0.1 ± 3.91	0.0001
	Experimental	-12.5 ± 6.83	

## Discussion

This study aimed at examining the effects of a pranayama exercise on CA candidates’ anxiety. Findings revealed that after the study intervention, the level of anxiety in the experimental group decreased significantly. On the other hand, the level of anxiety in the control group also decreased even though the decrease was not statistically significant. The magnitude of the decrease in anxiety level in the experimental group was five times more than the decrease in the control group. Between-group differences were also statistically significant.

Tekur et al reported that their seven-day pranayama-asana yoga program reduced state anxiety among patients with chronic back pain by 16%.^22^ In addition, Satyapriya et al found that their integrated yoga program was effective in reducing pregnant women’ anxiety.^23^ Ramesh also found three-day pranayama and music therapy effective in alleviating pain and anxiety among patients undergoing cardiac surgery.^24^ Besides, Muniyandi reported the effectiveness of fifteen-minute pranayama in alleviating alcoholics’ anxiety^25^ and Mehrotra et al found that their three-month yoga program significantly alleviated healthy young people’s anxiety.^26^

The results of all aforementioned studies are in agreement with the findings of the present study. However, in these studies, pranayama was used in combination with other yoga exercises and in long periods of time while in the present study, only sukha pranayama was implemented for five minutes. There are a wide range of pranayama exercises. The protocols and lengths of these exercises are different from each other.^27^ For instance, sukha pranayama is a slow and rhythmical type of breathing which is believed to increase the sensitivity of baro-reflexes and decrease the activity of chemical reflexes. Besides, it reduces systolic and diastolic blood pressures and heart rate among patients with hypertension. It is also effective in managing anxiety disorders through weakening cardiac autonomic responses.^13^

The findings of the present study indicated that although patients’ anxiety alleviated at T2, it increased in both groups at T3, i.e. before experiencing CA. According to Eng et al, significant increases in pre-CA anxiety can exacerbate patients’ physical symptoms and affect the prognosis of their conditions.^28^ Therefore, anxiety management modalities need to be used as close as possible to the time of CA. It is noteworthy that none of the previous studies had investigated the long-term effects of pranayama on anxiety and hence, conducting further studies is recommended.

Personal characteristics and appearance of healthcare professionals can affect patients’ anxiety. Slight decrease in the anxiety score of the patients in the control group can be attributed to the supportive role of nurses and the effects of their relationship with and presence for the patients. Given the potential effects of different data collection techniques, future studies are recommended to evaluate patients’ anxiety through using different anxiety assessment scales in combination with assessing their physiological and clinical parameters.

## Conclusion

Based on the findings of the current study, sukha pranayama is effective in alleviating CA candidates’ anxiety. Therefore, using this simple and easy-to-use non-invasive non-pharmacological technique is recommended to nurses for managing CA candidates’ anxiety. Alongside with growing public interest in complementary and alternative therapies, nurses need to receive educations about the concepts and the principles of using these therapies. Complementary therapy research centers and organizations can develop programs to provide such educations to nurses and empower them for using the therapies in their daily practice.

## Ethical approval

The Research Center and the Ethics Committee of Kashan University of Medical Sciences, Kashan, Iran approved the study (the approval code: IR.KAUMS.REC1394.84). We secured the permission of the administrators of the study setting. All participants enjoyed the latitude to either agree or disagree with the participation in the study or withdraw from it. Informed consent was obtained from all participants and they were ensured about the confidential management of their data.

## Competing interests

Authors declare no conflict of interest in this study.
